# Trigeminal Proprioception Evoked by Strong Stretching of the Mechanoreceptors in Müller's Muscle Induces Reflex Contraction of the Orbital Orbicularis Oculi Slow-Twitch Muscle Fibers

**Published:** 2014-08-12

**Authors:** Kiyoshi Matsuo, Ryokuya Ban, Midori Ban, Shunsuke Yuzuriha

**Affiliations:** Department of Plastic and Reconstructive Surgery, Shinshu University School of Medicine, Matsumoto, Japan

**Keywords:** orbicularis oculi reflex, trigeminofacial reflex, slow-twitch fibers, mechanoreceptors in Müller's muscle, blepharospasm

## Abstract

**Objective:** The mixed orbicularis oculi muscle lacks an intramuscular proprioceptive system such as muscle spindles, to induce reflex contraction of its slow-twitch fibers. We evaluated whether the mechanoreceptors in Müller's muscle function as extrinsic mechanoreceptors to induce reflex contraction of the slow-twitch fibers of the orbicularis oculi in addition to those of the levator and frontalis muscles. **Methods:** We evaluated in patients with aponeurosis-disinserted blepharoptosis whether strong stretching of the mechanoreceptors in Müller's muscle from upgaze with unilateral lid load induced reflex contraction of the orbicularis oculi slow-twitch fibers and whether anesthesia of Müller's muscle precluded the contraction. We compared the electromyographic responses of the bilateral orbicularis oculi muscles to unilateral intraoperative direct stimulation of the trigeminal proprioceptive nerve with those to unilateral transcutaneous electrical stimulation of the supraorbital nerve. **Results:** Upgaze with a unilateral 3-g lid load induced reflex contraction of the bilateral orbicularis oculi muscles with ipsilateral dominance. Anesthesia of Müller's muscle precluded the reflex contraction. The orbicularis oculi reflex evoked by stimulation of the trigeminal proprioceptive nerve differed from that by electrical stimulation of the supraorbital nerve in terms of the intensity of current required to induce the reflex, the absence of R1, and duration. **Conclusions:** The mechanoreceptors in Müller's muscle functions as an extramuscular proprioceptive system to induce reflex contraction of the orbital orbicularis oculi slow-twitch fibers. Whereas reflex contraction of the pretarsal orbicularis fast-twitch fibers functions in spontaneous or reflex blinking, that of the orbital orbicularis oculi slow-twitch fibers may factor in grimacing and blepharospasm.

The levator muscle consists of nonskeletal fast-twitch fibers and skeletal slow-twitch fibers,^1^ whose neuromuscular units for contraction are separated (Fig 1a).^2^^,^^3^ The nonskeletal fast-twitch fibers of the levator and superior rectus muscles are voluntarily contracted by excitation of the rostral interstitial nucleus of the medial longitudinal fasciculus, the interstitial nucleus of Cajal, the M-group, and the oculomotor neurons for vertical gaze control.^4^^,^^5^ Because the frontalis and orbicularis oculi muscles consist of skeletal fast-twitch and slow-twitch fibers (Fig 1a),^6^^,^^7^ the neuromuscular unit for contraction of each fiber type differs as well.^2^^,^^3^ The skeletal fast-twitch fibers of the frontalis and orbicularis oculi muscle are voluntarily contracted by excitation of the primary motor cortex and facial motor neurons. Whereas mixed limb skeletal muscles have intrinsic muscle spindles that are required to induce reflex contraction of their slow-twitch fibers owing to proprioception evoked by stretching of mechanoreceptors in the muscle spindles,^8^ the mixed levator, frontalis, and orbicularis oculi muscles seemingly lack these intrinsic muscle spindles.^9^^-^11 Accordingly, a specialized proprioceptive system to contract the levator, frontalis, and orbicularis oculi slow-twitch muscle fibers is believed to be present outside the muscles.

The supratarsal Müller's muscle is located between the levator muscle and the tarsus (Fig 1a) and is innervated by sparse sympathetic fibers, the interstitial cells of Cajal, and abundant myelinated trigeminal proprioceptive fibers in a palisade arrangement as mechanoreceptors.^11^^-^13 The trigeminal proprioceptive nerve fibers in Müller's muscle converge as a transverse nerve on the proximal aspect of the muscle, join into the lacrimal branch of the ophthalmic trigeminal nerve, pass through the superior orbital fissure and trigeminal ganglion, and reach the mesencephalic trigeminal nucleus to possibly connect with the locus ceruleus through gap junctions (Fig 1).^11^^,^^14^

We have reported that voluntary contraction of the levator fast-twitch muscle fibers stretches the mechanoreceptors in Müller's muscle to evoke trigeminal proprioception, which induces reflex contraction of the levator and frontalis slow-twitch muscle fibers to involuntarily raise the eyelid and eyebrow against gravity (Fig 1a),^15^^-^20 and that a hydraulic mechanism caused by trauma to the globe impairs trigeminal proprioceptive evocation, which reduces reflex contraction of the levator and frontalis slow-twitch muscle fibers, resulting in eyelid and brow ptosis.^21^ In addition, we have described that unilateral direct electrical stimulation to the trigeminal proprioceptive fibers induces a phasic short-latency monosynaptic response in the ipsilateral levator slow-twitch muscle fibers (Fig 1a).^22^ Such stimulation also produced a phasic short-latency monosynaptic response in the ipsilateral frontalis muscle and prolonged long-latency polysynaptic responses in the bilateral frontalis slow-twitch muscle fibers with ipsilateral dominance as well as prolonged long-latency polysynaptic responses in the orbicularis oculi muscles (Fig 1a).^23^

Under these circumstances, we hypothesized that the mechanoreceptors in Müller's muscle functioned as extrinsic mechanoreceptors that induced reflex contraction of the orbicularis oculi slow-twitch fibers in addition to the levator and frontalis slow-twitch fibers via the trigeminal proprioceptive neurons in the mesencephalon (Fig 1). Whereas the levator and frontalis muscles function as eyelid-opening muscles, the orbicularis oculi muscle serves as an eyelid-closing muscle. It therefore seems controversial that 2 opposing neural circuits are stimulated by the same trigeminal proprioception. However, electrical stimulation to the trigeminal proprioceptive fibers that innervate the mechanoreceptors in Müller's muscle may indeed activate the neuromuscular unit for contraction of the orbicularis oculi slow-twitch fibers, which mainly exist in the orbital portion and do not directly antagonize eyelid opening (Fig 1).^6^^,^^7^^,^^24^

To prove our hypothesis regarding reflex contraction of the orbicularis oculi slow-twitch muscle fibers, we first evaluated whether upgaze with metal weight loading on the pretarsal skin induced reflex contraction. We then evaluated whether intraoperative direct electrical stimulation of the trigeminal proprioceptive fibers innervating the mechanoreceptors in Müller's muscle induced reflex contraction of the orbicularis oculi slow-twitch muscle fibers in comparison with the blink reflex.

## PATIENTS AND METHODS

### Patients

**Figure 1:**
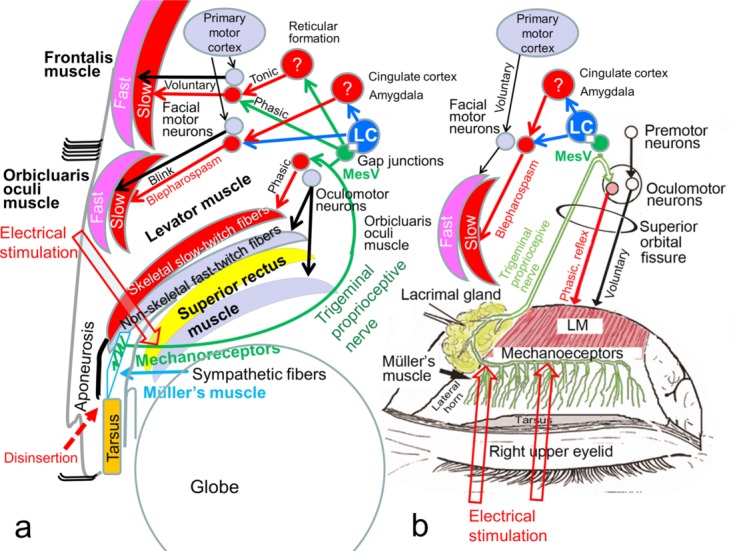
Neuroanatomy for the contraction of the levator, frontalis, and orbicularis oculi fast-twitch and slow-twitch muscle fibers. (*a*) Sagittal view. Black arrows indicate voluntary contraction of the levator, frontalis, and orbicularis oculi fast-twitch muscle fibers. Red arrows indicate involuntary reflex contraction of the levator, frontalis, and orbicularis oculi fast-twitch muscle fibers. Green arrows indicate the proprioceptive nerve. Disinsertion indicates that the levator aponeurosis is disinserted from the tarsus. (*b*) Frontal view for the electrical stimulation of the trigeminal proprioceptive nerve. Question marks indicate unknown nucleus at the reticular formation, cingulate cortex, or amygdala. Fast indicates fast-twitch muscle fibers; Slow, slow-twitch muscle fibers; mesV, mesencephalic trigeminal nucleus; LC, locus ceruleus; Phasic, phasic contraction; Reflex, reflex contraction; Tonic, tonic contraction.

One hundred Japanese patients with aponeurosis-disinserted blepharoptosis (85 women and 15 men; aged 55.9 ± 9.3 years) were enrolled for upgaze with lid loading and intraoperative electrical stimulation studies.^15^^,^^16^ The disinserted aponeurosis (Fig 1a) in all 100 patients was intraoperatively confirmed and fixed to the tarsus.^15^^,^^16^ The study protocol was approved by our institutional review board for human subjects. All patients were fully informed about the nature of the study and gave their written consent for participation.

### Upgaze with lid load for increased stretching of the mechanoreceptors in Müller's muscle

Before upgazing, 2 to 3 drops of 0.4% oxybuprocaine hydrochloride were administered on the surface of the cornea to prevent corneal reflex. The unilateral eyelid of the dominant eye in 100 patients was loaded with a 3-g metal weight on the pretarsal skin using double-sided adhesive tape. The patients were instructed to maintain a 60-degree upward gaze toward a corresponding target marked on the wall (Fig 2a). For subjects in whom involuntary contraction occurred in the orbicularis oculi muscles, we administered 4% lidocaine to anesthetize the mechanoreceptors in Müller's muscle. These patients were made to lie in a supine position, raise their chins, and gaze downward. The upper eyelids of the dominant eyes were detached from the globes with a small retractor for 60 seconds to create a space in the upper fornix. Four to 5 drops of 4% lidocaine were administered into the space and were retained in this position by gravity to exclusively anesthetize the mechanoreceptors in Müller's muscle. The 3-g metal weight was then again loaded on the pretarsal skin and the patients were asked again to reestablish a 60-degree upward gaze to evaluate if involuntary contraction of the orbicularis oculi muscles was present (Fig 2b).

### Intraoperative electrical stimulation of the trigeminal proprioceptive nerve

We compared the electromyographic responses of the bilateral orbicularis oculi muscles to unilateral transcutaneous electrical stimulation of the supraorbital nerve for the blink reflex with those to the trigeminal proprioceptive nerve in 9 of 100 patients who gave their informed consent for this study. Before electrical stimulation of the trigeminal proprioceptive nerve, the corneal surface and surfaces of the levator muscle and aponeurosis were anesthetized with 2 to 3 drops of 0.4% hydrochloride and local injection of 5 to 10 mL of 1% lidocaine with epinephrine in each eyelid.

**Figure 3:**
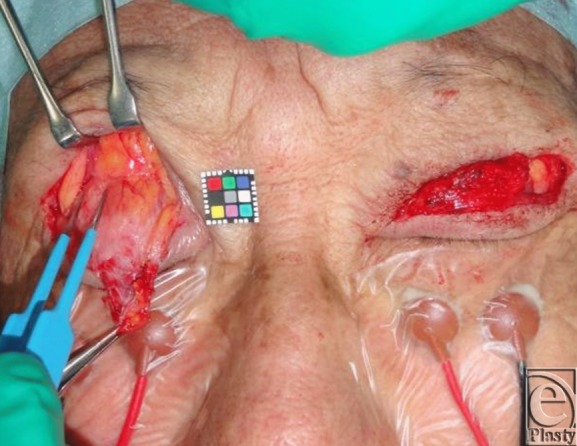
Intraoperative electrical stimulation of the trigeminal proprioceptive nerve innervating the mechanoreceptors in Müller's muscle in a 61-year-old woman with aponeurosis-disinserted aponeurosis. A forceps device is electrically stimulating the trigeminal proprioceptive nerve, which runs transversely between the distal levator muscle belly and the proximal Müller's muscle (refer to Fig 1b). The electrodes on the orbital orbicularis oculi muscles are recording responses.

Electrophysiological activity of the orbicularis oculi muscles was recorded using Ag-AgCl surface electrodes that were 8 mm in diameter and filled with impedance-reducing paste (Fig 3). The active electrode was placed over the lateral one-third of the orbital portion of the orbicularis oculi muscle and the reference electrode was placed 2 cm medially to the active electrode (Fig 3). The ground electrode was attached to the subject's chin. Electrical stimulation of the supraorbital nerve for measurement of orbicularis oculi reflexes was delivered using a pair of surface electrodes according to the guidelines of the international federation of the clinical neurophysiology.^25^ Electrical stimulation of the transverse trigeminal proprioceptive nerve on the proximal Müller's muscle was delivered using a forceps-type electrical stimulator. The cathode was placed beneath the center of the upper margin of the Müller's muscle, and the anode was placed near the lacrimal gland to avoid directly stimulating the orbicularis oculi muscle (Figs 1b and 3). To accurately obtain an orbicularis oculi reflex, a single electrical stimulus to the transverse trigeminal proprioceptive nerve required a constant 0.1-milisecond current pulse of 200 μV and 14.29 ± 6.73 mA, while that to the supraorbital nerve needed a constant 0.1-milisecond current pulse of 200 μV and 6.57 ± 2.70 mA. An interval of at least 7 seconds between electrical stimuli was chosen to avoid habituation of the blink reflex. All electromyographic recordings were made using an electromyograph (Neuropack 8; Nihon-Kohden Tokyo, Japan) with a band pass frequency filter set at 20 to 3000 Hz. Five trials were performed to detect the location of the transverse trigeminal proprioceptive nerve on the proximal Müller's muscle. Five electromyograms with larger amplitudes of the responses in the orbicularis oculi muscles were superimposed, and the shortest latency among the responses and the duration of the responses were calculated.

Because muscle responses were induced bilaterally, we compared the latencies and durations of ipsilateral responses. Data were analyzed using the Mann-Whitney *U* test with SPSS software (IBM). A *P* value of less than 0.05 indicated a statistically significant difference.

## RESULTS

Upgaze with a unilateral 3-g lid load induced involuntary contraction of the bilateral orbicularis oculi muscles with ipsilateral dominance in 13 of 100 patients (Fig 2a). After anesthesia of the mechanoreceptors in Müller's muscle in these patients, repeated upgaze with a unilateral 3-g lid load did not result in involuntary contraction (Fig 2b).

**Figure 4:**
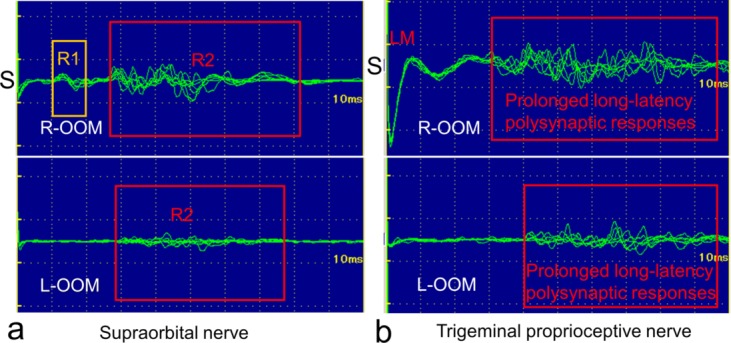
Representative orbicularis oculi reflexes induced by electrical stimulation of the supraorbital nerve (*a*) and the trigeminal proprioceptive nerve innervating the mechanoreceptors in Müller's muscle (*b*) in a 61-year-old woman, as shown in Figure 3. S indicates the side of electrical stimulation; R-OOM, right orbicularis oculi muscle; L-OOM, left orbicularis oculi muscle; LM, a volume-conducted response from the ipsilateral levator muscle to orbicularis oculi muscle.

**Figure 5:**
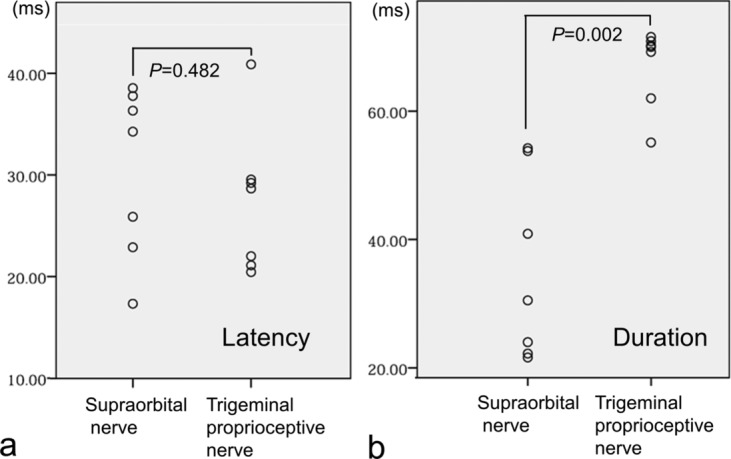
Statistical comparison of the latency (*a*) and duration (*b*) of the orbicularis oculi reflexes between electrical stimulation of the trigeminal proprioceptive nerve and that of the supraorbital nerve.

Stimulation of the supraorbital nerve induced an R1 response in the ipsilateral orbicularis oculi muscle and R2 responses (mean latency: 30.4 milliseconds; mean duration: 35.3 milliseconds) in the bilateral orbicularis oculi muscles with ipsilateral dominance (Fig 4a). Stimulation of the trigeminal proprioceptive nerve on the proximal Müller's muscle did not induce a phasic oligosynaptic response but rather induced a short-latency phasic response as a volume-conducted response from the ipsilateral levator muscle to the orbicularis oculi muscle^22^ as well as prolonged long-latency polysynaptic responses (mean latency: 27.4 milliseconds; mean duration: 67.0 milliseconds) in the bilateral orbicularis oculi muscles with ipsilateral dominance (Fig 4b). The mean latency of prolonged long-latency polysynaptic responses (27.4 milliseconds) induced by stimulation of the trigeminal proprioceptive nerve did not significantly differ from that of R2 responses (30.4 milliseconds) induced by stimulation of the supraorbital nerve (*P* = 0.482) (Fig 5a). In contrast, the mean duration of prolonged long-latency polysynaptic responses (67.0 milliseconds) induced by stimulation of the trigeminal proprioceptive nerve was significantly longer than that of R2 responses (35.3 milliseconds) induced by stimulation of the supraorbital nerve (*P* = 0.002) (Fig 5b).

## DISCUSSION

Evinger et al^26^ and Gruart et al^27^ reported that electromyographic activity in the orbicularis oculi muscles did not change according to vertical gaze movements. Upgaze with a 3-g lid load did not increase touch, pain, or temperature sensation but did increase proprioception evoked by stretching of the mechanoreceptors in Müller's muscle. In patients with aponeurosis-disinserted blepharoptosis,^15^^,^^16^^,^^28^ the mechanoreceptors in Müller's muscle are sensitized to enhance reflex contraction of the levator and frontalis slow-twitch muscle fibers for maintenance of an adequate visual field. Since upgaze with a 3-g lid load did not induce a visible reflex contraction of the orbicularis oculi muscles in any patient and local anesthesia of the Müller's muscle precluded involuntary contraction of the orbicularis oculi muscles in all 13 patients tested, the presence of strong stretching of the mechanoreceptors in Müller's muscle appeared to evoke strong trigeminal proprioception that induced reflex contraction of the bilateral orbicularis oculi slow-twitch fibers.

The orbicularis oculi reflex elicited by electrical stimulation of the trigeminal proprioceptive nerve differed from that by electrical stimulation of the supraorbital nerve in terms of the intensity of electrical current required to induce the reflex (14.29 mA vs 6.57 mA), the absence of an R1 response, and the duration of the prolonged long-latency polysynaptic responses (67.0 milliseconds vs 35.3 milliseconds). Meanwhile, the electrical current needed to consistently induce reflex contraction of the levator slow-twitch fibers by direct electrical stimulation of the trigeminal nerve was found to be 3 mA,^22^ while that for reflex contraction of the frontalis slow-twitch fibers by transcutaneous electrical stimulation of the trigeminal proprioceptive nerve was 15 mA.^23^ Consequently, weak stretching of the mechanoreceptors in Müller's muscle in primary gaze with weak proprioceptive evocation may induce reflex contraction of the levator slow-twitch muscle fibers, moderate stretching of the mechanoreceptors in Müller's muscle in upward gaze with moderate proprioceptive evocation may enhance reflex contraction of the levator slow-twitch muscle fibers and evoke reflex contraction of the frontalis slow-twitch muscle fibers, and strong stretching of the mechanoreceptors in Müller's muscle with strong proprioceptive evocation may induce reflex contraction of the orbicularis oculi slow-twitch muscle fibers (Fig 1).

Because the corneal surface was anesthetized, reflex contraction of the orbicularis oculi slow-twitch fibers induced either by stretching of the mechanoreceptors in Müller's muscle or by electrical stimulation of the trigeminal proprioceptive nerve could be distinguished from the corneal reflex with contraction of the orbicularis oculi fast-twitch muscle fibers,^29^ which may have possibly been induced by electrical stimulation or mechanical stimulation of the cornea with the weight-loaded eyelid.

The orbicularis oculi muscle consists of 3 distinct concentric units: the pretarsal, preseptal, and orbital portions. The pretarsal portion is almost completely composed of fast-twitch fibers, whereas the preseptal portion contains 10% to 20% slow-twitch fibers.^30^ The orbital portion has more slow-twitch fibers than both other reegions^7^ and forms an extrinsic system by blending with the corrugator supercilii, procerus, and frontalis muscles, which also contain high percentages of slow-twitch fibers.^7^^,^^31^^,^^32^ The fast-twitch fibers of the pretarsal and preseptal orbicularis oculi muscle are involved in phasic movements such as spontaneous and reflex blinking and mild eyelid closure. Meanwhile, the slow-twitch fibers of the orbital orbicularis oculi muscle are involved in tonic postural movements and serrated eyelid closure, which recruits the orbital portion with the corrugator supercilii and procerus muscles.^6^^,^^33^ In myotonic dystrophy with atrophy of skeletal slow-twitch fibers, although electrical stimulation of proprioceptive nerves in mixed limb skeletal muscles does not induce reflex contraction of slow-twitch muscle fibers (ie, the Hoffmann reflex), electrical stimulation of the supraorbital nerve induces reflex contraction of the orbicularis oculi fast-twitch muscle fibers (R2) as a blink reflex.^8^ Electrical stimulation of the trigeminal proprioceptive nerve was found to activate the neuromuscular unit for contraction of the levator and frontalis slow-twitch muscle fibers.^22^^,^^23^ Similarly, this appeared to stimulate the neuromuscular unit for contraction of the orbicularis oculi slow-twitch muscle fibers as well. The orbicular oculi reflex induced by electrical stimulation of the trigeminal proprioceptive nerve may thus resemble grimacing or blepharospasm with contraction of slow-twitch fibers rather than a blinking reflex with contraction of fast-twitch muscle fibers.

Since crow's feet in the elderly can be flattened by injection of botulinum toxin type A, they are recognized as hyperkinetic facial wrinkles,^34^ that indicate the presence of increased tonic contraction of the orbital orbicularis oculi slow-twitch muscle fibers. In elderly individuals whose levator aponeurosis is considerably disinserted from the tarsus,^16^^,^^28^^,^^35^ the mechanoreceptors in Müller's muscle might be strongly stretched in primary gaze to induce reflex contraction of the orbital orbicularis oculi slow-twitch fibers accompanied with the corrugator supercilii, and porcerus slow-twitch muscle fibers. 7^,^^31^^,^^32^ The resulting slightly grimacing face is often encountered in elderly people.

The cortical control of eyelid closure is not well understood.^36^ Only relatively recently has there been the finding that a major source is the cingulate cortex as well as lesser sources in primary motor cortex.^37^ This was first defined in the primate, and then confirmed in humans with both transcranial magnetic stimulation mapping^38^ and functional magnetic resonance imaging.^39^ New findings show a major input from the amygdala which presumably plays a role in behaviors such as emotional facial expressions.^40^ The locus ceruleus, which possibly connects with the mesencephalic trigeminal nucleus through gap junctions (Fig 1),^14^ projects ascending axons to the forebrain, the cingulate cortex, the amygdala, and the spinal motoneurons to facilitate muscle tone by involuntary contraction of skeletal slow-twitch muscle fibers.^41^^-^44 The locus ceruleus has been also reported to densely project to the facial motor neurons as well,^45^^,^^46^ and this projection appears to be excitatory since extracellular microiontophoretic application of noradrenaline increases the activity of these motoneurons.^47^^-^50 Together with our results, it can be interpreted that serrated eyelid closure with reflex contraction of the orbicularis oculi slow-twitch muscle fibers may be caused by trigeminal proprioception evoked from strong stretching of the mechanoreceptors in Müller's muscle via excitation of the mesencephalic trigeminal nucleus, the locus ceruleus, the cingulate cortex, or the amygdala, whereas mild eyelid closure with voluntary contraction of the orbicularis oculi fast-twitch muscle fibers may be caused by excitation of the primary motor cortex (Fig 1). Increased involuntary contraction of the orbital orbicularis oculi slow-twitch muscle fibers is always observed in yawning for arousal,^51^^,^^52^ which consist of opening the mouth wide and involuntary serrated eyelid closure. Increased contraction of the extraocular muscles, including the levator nonskeletal fast-twitch muscle fibers may retract the globes backwards and strongly stretch the mechanoreceptor in Müller's muscle to stimulate the locus ceruleus, which increases wakefulness and induces involuntary serrated eyelid closure by reflex contraction of the orbicularis oculi slow-twitch fibers.

## CONCLUSIONS

Trigeminal proprioception evoked both by strong stretching of the mechanoreceptors in Müller's muscle from upgaze with lid load and by electrical stimulation of the trigeminal proprioceptive nerve innervating the mechanoreceptors in Müller's muscle induces reflex contraction of the orbital orbicularis oculi slow-twitch muscle fibers not for blinking, but rather for grimacing or blepharospasm. Similarly to the levator and frontalis muscles, the orbicularis oculi muscle appears to possess an extramuscular proprioceptive system, that is, the mechanoreceptors in Müller's muscle, which is activated by strong stretching of the mechanoreceptors owing to contraction of the levator and superior rectus nonskeletal fast-twitch fibers. The next steps in this investigation are to differentiate blepharospasm from blink movements and to apply the findings to surgical control of grimacing in elderly people and blepharospasm.

## Figures and Tables

**Figure 2:** Upgaze with a 3-g weight loading before (*a*) and after (*b*) anesthesia of Müller's muscle in a 58-year-old woman with aponeurosis-disinserted blepharoptosis.
